# Male Psyllids Differentially Learn in the Context of Copulation

**DOI:** 10.3390/insects8010016

**Published:** 2017-02-07

**Authors:** Dara G. Stockton, Xavier Martini, Lukasz L. Stelinski

**Affiliations:** 1 Citrus Research and Education Center, Department of Entomology and Nematology, University of Florida, Lake Alfred, FL 33850, USA; stelinski@ufl.edu; 2 North Florida Research and Education Center, Department of Entomology and Nematology, University of Florida, Quincy, FL 32351, USA; xmartini@ufl.edu

**Keywords:** insect learning, Asian citrus psyllid, olfaction, mate choice, olfactometry, EAG

## Abstract

In the Asian citrus psyllid, *Diaphorina citri* Kuwayama, stimulatory cuticular hydrocarbons act as sex pheromone attractants. Male psyllids locate aggregations of females using those olfactory cues, as well as vibrational communication on the plant surface. Although previous research has indicated that learning plays a role in modulating female reproductive behaviors in psyllids, it is unknown whether males similarly use learning to increase the likelihood of copulatory success. We used an olfactometer-based bio-assay to study the effects of experience on male response to female odor. First, we compared male attraction to female odor in virgin and previously mated males. Second, we tested the effect of several modes of experience with a novel odor, vanillin, to determine whether mating, feeding, or general environmental exposure elicited a learned response. We found that male attraction to female odor significantly increased after mating experience. In addition, we found that males learn about odor specifically in the context of mating, rather than feeding or general exposure. Electrophysiological measurements of antennal response to odorants confirmed that mating status did not affect the sensitivity of the peripheral nervous system to volatile stimuli implicating learning at the level of the central nervous system. These results suggest that male response to female odor is not an entirely innate behavior. Males may require mating experience with female conspecifics to develop attraction to those olfactory cues produced by the female and in association with the female’s habitat. This adaptive plasticity may allow males to detect females in an ever-changing environment and promote diversification and further specialization on different host genotypes.

## 1. Introduction

The Asian citrus psyllid, *Diaphorina citri* Kuwayama, is a sap-sucking hemipteran that transmits *Candidatus* Liberibacter asiaticus, the putative causal agent of huanglongbing [1,2,3]. In an effort to slow the spread of the disease in citrus growing regions worldwide, considerable research on the behavior and ecology of *D. citri* has focused on the reproductive biology and mate finding behavior of this devastating agricultural pest species. Psyllids are known to use multi-modal communication to detect mates within a complex host plant environment [4,5]. Vibrational communication is widespread among Hemiptera [6,7,8] and in addition to functioning as an independent communication method, appears to modulate other forms of communication including long-range attraction and short-range courtship pheromone production [5,8,9].

In the southern green stink bug, *Nezara viridula*, male-produced sex pheromones first attract conspecifics to a host plant, then vibrational signals are used to specifically locate mates along the plant surface [9,10]. Similarly, psyllids appear to use both forms of communication to locate mates within the complex host plant environment [11]. During migration or movement between hosts, *D. citri* first uses herbivore-induced plant response volatiles, and the release of methyl-salicylate, to locate aggregations of conspecifics [12]. Once on the host plant, substrate-borne vibrational communication is used by male *D. citri* to call to females on the plant surface via rapid wing movements [4,11]. Females then reply, facilitating their location by males on the host plant [11,13]. This appears common among most psyllid species [5] although the characteristics of their vibrational calling is likely species specific [14]. In an effort to exploit the dueting behaviors of reproductive *D. citri*, artificial female calling devices are being developed for use in the field as a form of mating disruption [13,15,16,17].

Once in close proximity, female-produced cuticular hydrocarbons, which appear to function as sex pheromones, attract males to engage in courtship and copulation [12,18,19]. It is likely that these pheromones function as short-distance cues used after vibrational communication has allowed long-distance detection along the plant surface. At least three other psyllid species are known to use female produced sex-pheromones to attract mates including *Cacopsylla bidens* [5], *C. pyricola* [20], and *Bactericera cockerelli* [21]. However, there is some evidence to suggest that different psyllid species use semiochemicals in a manner that is species specific [5]. In male *C. pyricola*, cuticular hydrocarbon pheromones direct male-male aversion behaviors as well as male-female attraction [5,20]. In *D. citri*, however, it appears that the use of cuticular hydrocarbon semiochemicals is unidirectional to facilitate male-female attraction [22]. Males of this species do not display aversion to the odor of other males.

Research on the biology of psyllid reproduction and olfactory sex attractants is of particular interest due to the potential impacts on pest management practices. Recent advances in disease vector pest management have highlighted the importance of understanding the effects of experience on insect behavior [23,24,25]. Human disease vectors, such as mosquitoes [26,27,28,29,30], kissing bugs [31,32], tsetse flies [33,34], and sandflies [35], demonstrate ecologically significant learning events, driving host selection and oviposition site preferences via visual, olfactory, and even thermal stimulus experiences [23]. Similarly, *D. citri* behaviors, ranging from host preference to mate choice, may be guided by experience-dependent visual and olfactory associations with the host environment and conspecifics [36]. In human disease vectors, new techniques such as “zooprophylactic” measures aim to reduce harmful disease spread by exploiting the inherent learning mechanisms used by the vector to shift preference to non-human targets, such as cattle [37]. Similar approaches may be possible for phytopathogen vectors if more information is gathered about the nature of learning in these pest species.

With respect to the reproductive behavior of phytopathogen vectors such as *D. citri* it is important to determine whether male attraction to conspecific female odors is innate or experience-dependent. Contrary to the idea that sex pheromone responses are generally stable and not subject to changes with experience, there is evidence suggesting that sex pheromone responses can be surprisingly plastic [38,39]. Neurobiologically, exposure to sex pheromone is known to induce changes in the insect brain associated with olfactory recognition [39]. After frequently observing psyllid mating behaviors in the context of female mate choice, we hypothesized that males may learn about female odor such that male response to females is enhanced after early courtship and mating experiences. If male attraction is experience-dependent, it may facilitate population suppression technologies that manipulate male behavior with the purpose of disrupting mating. Indeed, there is sufficient evidence of male reproductive learning in other insect species to predict similar occurrences in male psyllids. In *Drosophila* sp., males discriminate among heterospecific and conspecific females through trial and error courtship experiences and young sexually immature males use courtship experience to refine their technique in lieu of future successful matings [40,41,42,43,44]. In the sweat bee, *Lasioglossum zephyrum*, males learn to avoid odors associated with unreceptive females [45], as well as closely related females due to selective pressures promoting outbreeding [46]. These learned responses to female traits appear to confer fitness benefits, where copulatory success is more likely in males that refine their ability to detect and court appropriate females.

To address this question in *D. citri*, we designed three experiments to test whether males learn about female odor, specifically in the context of mating, where copulation acts as a biologically significant unconditioned reinforcer. First, we compared the responses of mated and virgin males to female odor (proxy for volatile cuticular hydrocarbons thought to function as sex pheromone). If male attraction is experience-dependent, we expected only mated males to show preference for female odor. Then, we compared the acquisition of response of a novel olfactory stimulus, vanillin, in males exposed to the odor under different conditions: as an environmentally derived odor, an odor associated with females directly, and an odor associated with a food source. If the learned responses were explicitly linked to mating experience, we speculated that only the males exposed to vanillin associated with females directly should demonstrate a learned response. Finally, we compared the electrophysiological antennal responses of virgin and mated males to female whole body extracts to determine if changes in male response to female odor are caused by peripheral sensitization [39,47] or true learning.

## 2. Materials and Methods

### 2.1. Insect Colony

Insects were obtained from a greenhouse colony at the University of Florida, Citrus Research and Education Center campus in Lake Alfred, FL, USA [36]. Psyllids were collected from two to five year-old potted Valencia (*Citrus x sinesis*), orange jasmine (*Murraya paniculata*), or curry leaf plants (*Bergera koenigii*), maintained at 28 °C with a 14L:10D light cycle. Plants were rotated out of colony cages once per month to ensure plant health.

Virgin males were obtained weekly throughout the course of the study. Clippings from the general greenhouse colony were collected when there were aggregations of fourth to fifth instar nymphs. The clippings were transferred to cages with a single potted orange jasmine plant and monitored daily. Newly emerged adults were removed from the cages and sexed. Males were then re-released into a male only cage until use in experiments. To ensure the insects were reproductively mature [48], all males were one to two weeks old at the time of use. Males greater than two weeks old were discarded from the study.

Prior to use in the experiments described below, all psyllids were collected via manual aspiration from their respective colony cages. The psyllids were then removed individually from the collection vial with a paintbrush and carefully transferred to individual 1/4 dram glass vials with cork stoppers (Bio-Quip Products Inc., Rancho Dominguez, CA, USA). The psyllids were then sexed (in the case of the general colony collections for females) using a dissecting microscope, or were confirmed as males, and inspected for signs of poor health. Psyllids were discarded if they displayed grey-brown coloration, which has been linked to poor fecundity [49], or if they were abnormally small in size or showed physical injury due to handling. In this way, only healthy psyllids were used in the study.

### 2.2. Experiment 1

To test the effect of mating status on male response to female odor, we compared virgin and mated male response to female odor in a T-maze similar to that previously reported [50]. A vertically mounted, glass T-maze received charcoal filtered, humidified air pumped at 0.2 LPM (liters per minute) from a regulated flowmeter (ARS Inc., Gainesville, FL, USA). One of the chambers was baited with the experimental odor while the other arm remained an odorless control. To control for potential environmental effects, the apparatus was positioned within a white foam-board box. Positional bias was further monitored by rotating the T-maze 180° every 10 trials so that the target odor and control positions were rotated. The pump inputs were alternated every other repetition. Pump-line flow-rate was recorded at the start of every session. To remove the effects of individual tracking from the experiment, the T-maze itself was replaced every five trials (one psyllid per trial) with a clean piece of glassware. Previously, tracking has only been demonstrated in female *D. citri* [12,22]. Male psyllids do not appear to modify their behavior in response to olfactory stimuli produced from other males. However, we adopted a system of replacing the glassware after every five trials to avoid any potentially confounding effects of aggregation or dispersal [36]. A trial lasted 5 min (300 s) or until each psyllid made a selection. A selection was defined as chamber entry, 2 cm or more past the crux, for a minimum of 30 s. Psyllids were released into the apparatus from a 4 cm long inlet inserted into the base of the T-maze.

To provide female odor, five female psyllids were released into the odor source compartment of one T-maze chamber. The females were collected from the general colony. Only females with blue/green abdominal coloration were used. Female age and mating status was not standardized. Females were discarded if they were newly emerged, teneral adults; if they were grey/brown in coloration (a likely sign of poor health) or orange/yellow (indicating gravidity); appeared abnormally small or were otherwise injured. The other odor source lines pumped unscented air into the adjacent odor chamber. Virgin males (N = 48) were collected from the virgin male colony as previously described. Mated males (N = 52) were collected from a similar colony, although females were present and males were allowed to mate at will. All males were one to two weeks old at the time of use.

Four replicates of approximately 20 trials each were conducted. A trial consisted of a single psyllid. The number of trials varied between replicates due to variation in the number of available psyllids during the time of testing. One replication was performed per week for four weeks. All psyllids were euthanized after use and were not reintroduced into the colony.

### 2.3. Experiment 2

This experiment included four treatment groups in a randomized block design. Male psyllids were exposed to vanillin (CAS# 121-33-5, Sigma-Aldrich Corp., St. Louis, MO, USA) in one of four possible contexts: on a female, on a leaf disk, on the vial wall in which they were kept, or not at all. After 24 h, the males were removed and individually assayed for response to vanillin. Previous research indicated that vanillin was a suitable volatile for use in learning assays with *D. citri* [36,51].

In group 1 “Mating + Vanillin on Female,” (N = 47) previously unmated, virgin male psyllids were mated for 24 h with a female that was marked with a 10 μL vanillin-scented dot (50 μL 2.5% ethanolic vanillin/1 mL glue) on her thorax.

In group 2 “Mating + No Odor” (N = 40) males were mated with females marked with an unscented 10 μL dot (50 μL ethanol/1 mL glue) on the thorax. Group 2 acted as a direct control to group 1 and all psyllids in this group were considered vanillin naïve.

In group 3 “No Mating + Vanillin on Vial” (N = 47) males were exposed to the vanillin glue but not in the context of a female. It was applied to the interior vial wall. The same volume and vanillin concentration were used as in group 1. Males in this group were tested for response to vanillin after 24 h to control for the possibility that exposure alone leads to accentuated response to a novel odor.

In group 4, “No Mating + Vanillin on Leaf Disk” (N = 51) vanillin was paired with a nutritional reward (a leaf disk) to determine whether increased response to vanillin was due to pairing of the novel odor with females, or with a general reinforcing stimulus. The vanillin glue was applied to a leaf disk rather than a female. The same volume and vanillin concentration were used as in group 1 and group 3. In this way, male psyllids would encounter the stimulus in the context of feeding rather than mating. The same amount of glue, 10 μL, was applied to the leaf disks, the vials, and the females.

Preliminary observations suggested that 73% of psyllids mate within 2 h in confined conditions. For the two treatment groups paired with females, group 1 and group 2, one male and one female psyllid were placed in a 1/4 dram glass shell vial with a cork stopper for isolation. Paired insects were observed for 2 h to confirm mating. Unmated pairs were discarded from the study. For the other two treatment groups, group 3 and group 4, males were placed in a vanillin marked vial, or in a vial with a vanillin marked leaf disk for 24 h, respectively. Groups 3 and 4 did not have females present.

After 24 h, the males were removed from their individual vials and placed in new clean vials individually. They were then immediately used for testing in the Y-maze. All insects were used within 2 h of being removed from their “experience” vials. For the Y-maze behavior assay tests, one chamber of a glass Y-maze apparatus was baited with unscented air (25 μL 95% ethanol), while the other was baited with vanillin scented air (10 μL, 2.5% ethanolic vanillin; 15 μL 95% ethanol). The ethanol solutions were applied to a 2 cm cotton wick and were evaporated for 30 min prior to testing in a fume hood.

The Y-maze arms were baited the same way for all treatment groups. The Y-maze set-up was similar to that previously reported [36] and was similar to the T-maze. Only the shape of the assay glassware differed. Rather than a glass cylinder with two adjacent odor chambers, the Y-maze splits from a common base tube into a Y-shape. The two arms of the Y-maze received charcoal filtered, humidified air pumped at 0.2 LMP from a regulated flowmeter (ARS Inc.). A Y-maze was used in this experiment rather than a T-maze because it is a more conservative measure of behavior and provides the insects more distance from the inlet to the crux.

All of the assay controls were the same in experiments 1 and 2. The Y-maze was rotated every 10 trials, the glassware was replaced every five trials, and the pump inputs were rotated every other repetition. Psyllids were released into the apparatus from a 2 cm inlet inserted into the base of the Y-maze. Psyllids were excluded from the study if they failed to leave the inlet for 5 min, the duration of the trial. 

All four treatment groups were tested concurrently. Approximately 10–20 males from each treatment group were run in alternating groups of five on a single day. After five males from each treatment group were tested, another five from each group were run through the assay. The order of the treatment groups was varied for each replicate to ensure that time-of-day did not influence the results. A single day of recording comprised one replicate. The experiment was replicated three times. One replicate was performed per week for three weeks. The one-week time between replicates was necessary to procure mature virgin males, as described previously. During each replicate, each of the four treatments were set up 30 times, meaning 120 males were collected from the virgin colony and placed in individual vials. Of those, 10–20 males were tested in the T-maze per treatment per replicate due to overnight mortality or reduced health. Males were excluded that appeared weakened or unable to move properly through the Y-maze. We did not observe a difference in mortality among the four treatment groups.

### 2.4. Experiment 3

To determine whether male responses to female odor were affected by peripheral sensitization, we evaluated antennal responses by electroantennogram (EAG) of virgin and mated males. Two types of odorant stimuli were tested. First, whole body extracts from females at five concentrations were prepared as described below to measure male response to the female sex pheromone. Second, linalool (Aldrich Chemical Company, Milwaukee, WI, USA, 98% pure) was used as a known host plant volatile attractant of *D. citri* [52].

Whole body extracts were prepared as described in previous research [53]. Approximately 500 adult psyllid females were agitated in 500 μL of pentane for 10 min in a glass vial. Thereafter, the adults were removed and the solution was strained to remove particulate matter from the stock solution. Various concentrations of female whole body extracts were prepared with a serial dilution in increasing amounts of pentane. Prior to use in the EAG assay, pentane-based extracts were applied to 1.4 by 0.5-cm strips of Whatman No. 1 filter paper (Fisher, Pittsburgh, PA, USA) and solvent was allowed to evaporate under a fume hood for 30 min. The filter papers were then transferred to disposable glass Pasteur pipettes for use as stimulus cartridges in EAG assays. Linalool-loaded EAG cartridges were prepared identically to those described for extracted cuticular hydrocarbon volatiles by diluting neat chemical in pentane on a log scale.

EAG recordings were performed with an IDAC-2 acquisition controller connected to a universal single-ended probe (Type PRS-1) (Syntech, Kirchzarten, Germany) as described previously [4]. Antennal response was measured following stimulation with the whole body extract at five different concentrations. EAGs were recorded with silver wire electrodes fixed in glass micropipettes containing 0.5 M KCL. The recording electrode was positioned on the tip on one antenna, while the reference electrode was inserted into the back of the psyllid head. Charcoal-filtered, humidified airflow was provided through one arm of a modified glass Y-tube over the recording antenna by a regulated flowmeter (ARS Inc.) at 0.3 LPM. The Y-tube base was positioned 3mm from the antenna. Whole body extracts were manually introduced to the recording antenna through the opposite arm of the Y-tube using an attached 20 mL glass syringe. A 1 mL puff of air through the previously prepared stimulus cartridges delivered the odorant into the airstream of the Y-tube and onto the insect antenna.

The extracted cuticular hydrocarbon volatiles and linalool treatments were delivered in an ascending dosage order beginning with the solvent control per insect. Separate experiments were carried out with female whole body extract collections and linalool. All insects were euthanized after use and were not reused or rereleased in to the colony. We tested the antennal response in 10 virgin and 10 mated males to the extracted whole body wash. Then, another 10 virgin and 10 mated males were tested in response to linalool. EAG data were recorded using a Windows-based computer equipped with an interface card and software from Syntech. The computer was interfaced with a software-controlled amplifier and an A/D conversion circuit; it operated with 12-bit resolution.

### 2.5. Statistical Analysis

Comparisons of male responses in the olfactometer assays were analyzed with Chi-squared tests [36]. Within-group comparisons described differences in selection of a particular treatment group for arm A or arm B of the Y-maze. Between-group comparisons described differences in overall selection pattern between different treatment groups using a 2 × 2 chi-squared contingency table design. EAG data were analyzed with two-way analysis of variance and Fisher’s LSD (Least Significant Difference) tests [4]. Analyses were performed in R (Version 3.0.2; the R Foundation for statistical software R; Vienna, Austria).

## 3. Results

### 3.1. Experiment 1

**Figure 1:**
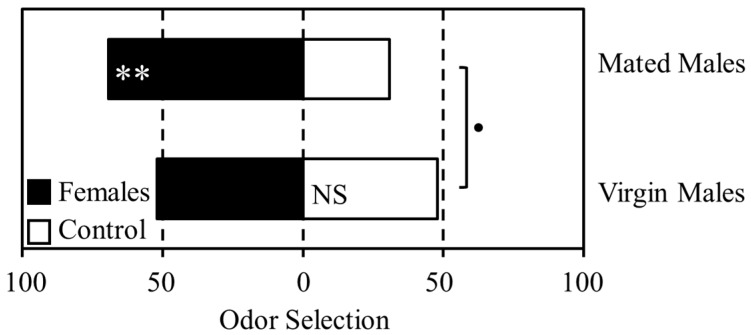
The effect of mating status on male response to female odor. Mated and virgin males were given a choice of selecting female odor (grey bars) or an unscented control (white bars) in a T-maze olfactometer. The *x*-axis indicates the percentage of psyllids selecting each odor source. Asterisks indicate statistically significant results; • <0.1, * ≤0.05, ** ≤0.01.

Mating status significantly affected male response to female odor in a T-maze olfactometer assay (Figure 1). Although there was no difference in selection between the female scented and control odor chambers for virgin males (Χ^2^_1_ = 0.08, *p* = 0.773), 69% of mated males selected the female scented chamber (Χ^2^_1_ = 7.69, *p* = 0.005). Comparison of overall selection pattern between virgin and mated males suggested a difference in response to female odor below the 0.1 probability level (Χ^2^_1_ = 3.08, *p* = 0.079), although this did not meet the generally accepted threshold of significance.

### 3.2. Experiment 2

**Figure 2:**
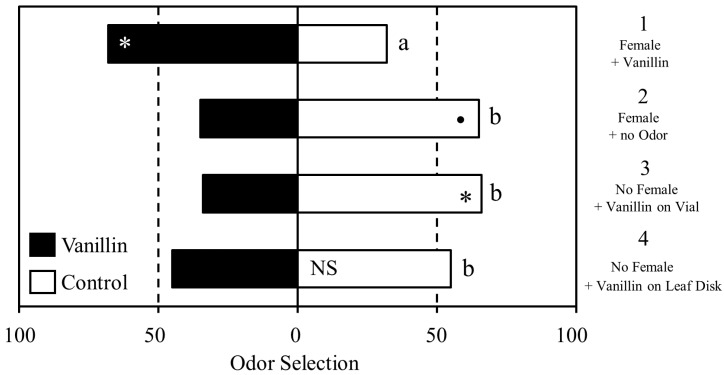
Learned response to vanillin depending on the conditioning environment. Significant within-group differences in selection the of vanillin (grey bars) or the unscented control arm (white bars) are indicated by asterisks; • <0.1, * ≤0.05. Significant differences in overall selection pattern between treatment groups are indicated by different letters.

Male *D. citri* displayed differences in response to vanillin based on the type of prior experience (Figure 2). Males in group 1 (vanillin on a female) showed a statistically significant increase in selection of the vanillin scented arm than the unscented arm (Χ^2^_1_ = 6.1489, *p* = 0.013). In contrast, males in group 2 (mated to an unscented female) appeared to display aversion to vanillin (Χ^2^_1_ = 3.6, *p* = 0.057), although it did not meet the generally accepted threshold of significance. Males in group 3 (vanillin on the vial wall) showed significant aversion to vanillin (Χ^2^_1_ = 4.79, *p* = 0.028). The insects in group 4 (vanillin on a leaf disk) showed no preference between the vanillin-scented and unscented arms (Χ^2^_1_ = 0.49, *p* = 0.483). Between-group comparisons revealed that group 1 differed significantly from group 2 (Χ^2^_1_ = 9.49, *p* = 0.002), group 3 (Χ^2^_1_ = 10.90, *p* = 0.001), and group 4 (Χ^2^_1_ = 5.25, *p* = 0.022) in overall selection pattern.

### 3.3. Experiment 3

**Figure 3:**
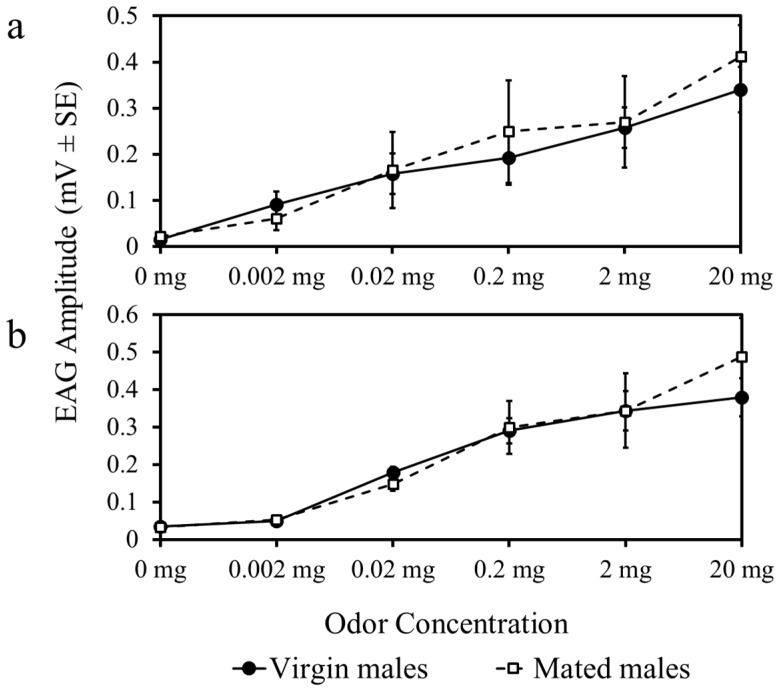
Antennal responses to female whole body extracts (**a**) and a stimulatory host-plant volatile, linalool (**b**), at increasing concentrations compared to an odorless blank (0 mg).

There were no differences in EAG response amplitude to whole body extracts (F_5,180_ = 0.81, *p* = 0.36) (Figure 3a) or linalool (F_5,180_ = 0.42, *p* = 0.21) (Figure 3b) among mated and virgin males. Response amplitude increased in virgin and mated male psyllids as the concentration of each odor was increased (Figure 3).

## 4. Discussion

Our data suggest that male attraction to female odor is not entirely an innate response. Rather, experience with receptive females enhances male attraction to odors associated with those females. Our data are consistent with previous research, which showed that only mated male *D. citri* display significant attraction to female odors [22]. However, at times our results were difficult to interpret. While female produced odors attract males in *D. citri*, they have not been strictly defined as pheromones and innate mate attraction to odors is relatively weak [19]. In addition, psyllid responses to olfactory stimuli are highly variable and difficult to study due to sensitivity to changes in barometric pressure and other environmental factors, which are difficult to control [54]. This may explain why, in at least two cases, our analysis revealed mild to insignificant differences between treatment groups, despite large differences within groups. Our findings are consistent with research on learned male mate choice among insects and some vertebrate species [40,41,43,44,45,55,56,57] and congruent with previous research in *D. citri* demonstrating behavioral plasticity in contexts other than reproduction, including feeding and host preference [36].

Our initial study found that male and female psyllids are capable of single stimulus visual and olfactory associations with the host plant, as well as complex compound conditioning tasks [36]. However, we did not rule out the possibility that male psyllids also learn in the context of mating, rather than host feeding alone as a form of reward. Our current data are the first to suggest that male *D. citri* preferentially associate environmental odors and/or female produced odors with receptive females. When conditioned to a novel olfactory stimulus in the presence of a food-based reward, the subsequent associative learning was lower than that observed with a copulation-based reward. Similarly, in the parasitoid wasp, *Leptopilina heterotoma*, female kairomone-host associations are strongest when oviposition is used as a reward [58]. Experience had the least effect on subsequent psyllid behavior when the novel odor was associated with the environment alone and lacked a reward. This suggests that the change in behavior observed in our studies reflects true associative learning, such that response shifts are not due to non-associative changes like sensitization or habituation [38,39,47,59].

In some insects, such as Lepidoptera, non-associative learning phenomena such as sensitization and habituation may account for changes in response to female pheromones [38,39,47,60]. In the oblique-banded leafroller, *Choristoneura rosaceana*, short-term pre-exposure to female pheromones is sufficient to reduce responsiveness in males [47], whereas in the cotton leafworm, *Spodoptera littoralis*, pre-exposure results in higher sensitivity to female pheromones [38]. To specifically test for the possibility of peripheral sensitization following mating, we used electrophysiological measurements of male antennal response (EAG) to odorants and confirmed no changes in male antennal sensitivity following mating. These results suggest that male experience resulted in true associative learning.

Male attraction to female odor is well documented in other psyllid species [5]. However, some of those studies have failed to account for the influence of experience on male preferences [61,62]. Males in these studies were commonly kept in cages with females, or were collected in the field. In other cases, virgin insects were used so an assessment of innate attraction is possible. In the potato/tomato psyllid, *B. cockerelli*, virgin males do strongly orient towards females despite a lack of experience, indicating an innate attraction to female-produced pheromones [20,21,63]. This is in contrast to our findings with *D. citri* and indicates species specificity with cues and behaviors, such that, along with the composition of vibrational signals across the plant surface, and the constituency of female-produced cuticular hydrocarbons, learning in psyllids is species specific. There are likely differences in the evolutionary pressure faced by each species that would encourage or discourage learned attraction, such a variation in resource availability across generations [64,65,66,67].

Evolutionarily, there are many potential benefits to selective and learned responses to conspecific pheromones [39,43,66,67]. Males may use acquired attraction to female odor to detect females in the complex host environment, especially when combined with other sensory modalities such as vibrational communication and visual detection. If experience increases the likelihood that males can locate females, then the likelihood of reproduction increases. Learning may also confer benefits with regard to avoiding predation [39]. When attention is focused on female pheromone perception, insects may display reduced perception of predator cues [68], although it is unknown if *D. citri* engages in active predator avoidance behaviors.

Another potential evolutionary benefit to male olfactory learning is that learning may facilitate some degree of mate choosiness in male psyllids [43]. In some species, mate choosiness in males is adaptive, where selection of females favors those with high fecundity and low risk of sperm competition [69,70]. Male mate choosiness may also take the form of predicting female receptivity [40], or avoiding misdirected matings with heterospecific species [41]. In this way, learning in the context of male mate selection may allow males to determine female quality such that resources are not divided among high quality and low quality mates [43]. To accomplish this, males must learn about the relative quality and availability of females in the given area, preferring high quality females based on innate preferences, previous experiences, and the abundance of mating opportunities available [69,71]. Research with *D. melanogaster* has shown that the number of successful matings increases in males with age and experience, presumably because male courtship behaviors and mate choosiness are refined over time [43]. However, because sex pheromone production and overall fertility of *D. melanogaster* increases with age, it is unclear to what extent learning contributes to mating success [72,73]. Future research is needed to determine if male *D. citri* use learning to refine interspecific and intraspecific discrimination among potential mates. Variation in female odor concentration and constituency, as well as abdominal color and size, may serve as potential variables males use to assess female quality.

It is interesting that a species such as *D. citri* would demonstrate learned preference for female odor, rather than entirely innate attraction. These insects are commonly described as having a promiscuous mating system, or scramble polygyny [74], although females are known to display clear choosiness and refuse the advances of some males [75,76]. Learning is not thought to be beneficial among males in such species because scramble competition relies on a series of rapid, indiscriminate encounters rather than careful selection among few partners. In addition, mate choosiness is specifically associated with certain male traits, which male psyllids appear to lack, including high male investment in the form of nuptial gifts or limited sperm availability [5,77]. Indeed, male psyllids are commonly observed attempting to mate with the first psyllid it encounters, regardless of the sex of the insect. However, we do not believe our data are incompatible with those hypotheses. While male psyllid mate choosiness appears greatly reduced compared to other species, learning in male psyllids may function to help detect females, and therefore maintains a beneficial function. Indeed, there is growing body of research on learning in the context of reproduction in *D. melanogaster* which indicates male learning is common and beneficial to insects in cases where promiscuity may be predicted [40,44,67].

It is our observation that in nature, male and female psyllids engage in practice courtship and copulation behaviors prior to the age in which they are reproductively mature, which occurs at three to four days post-eclosion [48]. It is common to see newly-eclosed, reproductively immature psyllids engage in courtship behaviors. It is during those early encounters that males may become familiar with female traits, specifically female-produced cuticular hydrocarbons. Combined with other forms of communication, early learned attraction to female odor may encourage selective courtship and maximize the number of successful matings. This would not be contradictory to a promiscuous approach to copulation, rather it would likely reduce the number of failed or misdirected mating attempts between males or heterospecific females [42]. In our experience, many mistaken mating attempts occur in artificial settings, such as inside a polystyrene Petri dish, wherein vibrational communication, that may otherwise help psyllids discriminate among potential mates, is compromised. Further research is needed to clarify the role of male mate choice in psyllids and whether male mate choice is refined by experience. It may also be necessary to elucidate the interaction between olfactory and vibrational communication in psyllids mate selection.

Our results may have implications for development of behaviorally based management tools for *D. citri* [22]. Semiochemical-based management tools for *D. citri* are currently under development [52]. Non-traditional control methods are also being explored, given the importance of this pest. If male attraction to female odor is experience-dependent, it may be possible to manipulate male behavior in such a way that mate detection and reproduction is suppressed. For example, appetitive learning in insects is specifically mediated by octopamine (RS-4-(2-amino-1-hydroxy-ethyl) phenol), a biogenic amine neurotransmitter found almost exclusively in invertebrate species [78]. While octopamine occurs in vertebrates, it does not appear to have a major role as compared with norepinephrine [79]. Indeed, appetitive learning is significantly suppressed in insects when octopamine antagonists are administered [80,81,82]. It therefore may be possible to introduce a novel form of mating disruption in agricultural settings by disrupting a target species’ ability to learn. While the potential for such application is distant and would require significant effort to develop a safe and target-specific method of application, pest control options expand with better understanding of the target species’ ecology and behavior.

## 5. Conclusions

Male *D. citri* become more responsive to the odors of receptive females following previous experience and a copulatory reward. This does not appear to be a peripheral effect; antennal sensitivity to both female-derived and host plant odorants did not change with male mating experience. Rather, the perceived significance of those odors likely changes after successful mating, increasing subsequent attraction towards conspecific females. Furthermore, olfactory learning in *D. citri* males appears to be selectively encouraged by copulatory rewards, where learning was not facilitated by nutritional reinforcement or environmental exposure alone. Such selective olfactory learning in the context of reproduction may allow *D. citri* males to more readily discriminate among heterospecific and conspecific females in the natural environment, and avoid potentially unfavorable matings with immature or otherwise unreceptive conspecific females.
